# Development and application of an LC-MS/MS method for 8 antiepileptic drugs and 2 metabolites using microsampling techniques (DBS and VAMS)

**DOI:** 10.1093/jat/bkaf073

**Published:** 2025-07-16

**Authors:** María Cobo-Golpe, Lucía Paniagua-González, Elena Lendoiro, Miriam Blanco-Ces, Ángela López-Rabuñal, Javier Abella, Dolores Castro, Cristina Melcón, Patricia Fuentes, Iria Carballeira, Carlos García, Carmen Gómez, Manuel López-Rivadulla Lamas, Angelines Cruz, Ana de-Castro-Ríos

**Affiliations:** Toxicology Service, Institute of Forensic Sciences, Universidade de Santiago de Compostela, Santiago de Compostela 15782, Spain; Toxicology Service, Institute of Forensic Sciences, Universidade de Santiago de Compostela, Santiago de Compostela 15782, Spain; Toxicology Service, Institute of Forensic Sciences, Universidade de Santiago de Compostela, Santiago de Compostela 15782, Spain; Toxicology Service, Institute of Forensic Sciences, Universidade de Santiago de Compostela, Santiago de Compostela 15782, Spain; Toxicology Service, Institute of Forensic Sciences, Universidade de Santiago de Compostela, Santiago de Compostela 15782, Spain; Neurology Service, University Hospital Complex of Ferrol, Ferrol 15405, Spain; Neurology Service, University Hospital Complex of Vigo, Vigo 36312, Spain; Pediatrics Service, University Hospital Complex of Vigo, Vigo 36312, Spain; Pediatrics Service, University Hospital Complex of Santiago de Compostela, Santiago de Compostela 15706, Spain; Pediatrics Service, University Hospital Complex of Ferrol, Ferrol 15405, Spain; Pediatrics Service, University Hospital Complex of Santiago de Compostela, Santiago de Compostela 15706, Spain; Pediatrics Service, University Hospital Complex of Santiago de Compostela, Santiago de Compostela 15706, Spain; Toxicology Service, Institute of Forensic Sciences, Universidade de Santiago de Compostela, Santiago de Compostela 15782, Spain; Toxicology Service, Institute of Forensic Sciences, Universidade de Santiago de Compostela, Santiago de Compostela 15782, Spain; Toxicology Service, Institute of Forensic Sciences, Universidade de Santiago de Compostela, Santiago de Compostela 15782, Spain

## Abstract

Therapeutic drug monitoring (TDM) of antiepileptic drugs (AEDs) is used for optimization and individualization of patients′ treatment. Capillary microsampling techniques are a promising alternative to conventional venous sampling for TDM. Both dried blood spots (DBS) and volumetric adsorptive microsampling (VAMS) devices are less invasive and patient-friendly sampling techniques which have been gaining interest in the last years. This study describes the development and validation of an LC-MS/MS method for the determination of 8 AEDs (Carbamazepine, Lacosamide, Levetiracetam, Lamotrigine, Phenobarbital, Valproic acid) and 2 metabolites (10,11-Dihydro-10-hydroxy-carbamazepine (DHCB) and carbamazepine-10,11-epoxide) in DBS and VAMS samples. The method was fully validated for linearity, selectivity, accuracy, precision, carryover, matrix effect, recovery and stability (15 days at room temperature and 72 h in autosampler). Moreover, the volume effect, volcano effect, and the hematocrit (Hct) effect were also assessed for DBS samples. All parameters showed satisfactory results, with a limit of quantification ranging from 0.5 to 10 µg/mL, depending on the analyte. Some instability issues were detected in DBS samples for oxcarbazepine. However, the inclusion of oxcarbazepine’s metabolite DHCB overcomes this problem as it was stable under both conditions tested. Moreover, this is the first DBS or VAMS method reporting the inclusion of DHCB, which seems essential for the TDM of oxcarbazepine. The method was applied to 80 paired samples from patients under treatment with these drugs in order to study the suitability of the method for the detection of these compounds, and compare concentrations in paired VAMS, DBS, whole blood and plasma samples. Ratios between paired samples show a promising correlation between microsampling techniques and plasma concentrations.

## Introduction

Therapeutic drug monitoring (TDM) is used routinely for antiepileptic drugs (AEDs) to optimize therapy because of the interindividual variability and narrow therapeutic ranges. Furthermore, in around 30% of the patients, multi-therapy is needed to achieve the best control of symptoms [1]. Traditionally, drug concentrations are measured in plasma or serum obtained by venous sampling by health professionals. This is a time consuming and stressful procedure, especially for children. As an alternative, in the latest years, new sampling methods have been considered for TDM of these drugs. Microsampling methods, such as Volumetric Absorption Microsampling (VAMS) devices or Dried Blood Spot (DBS) cards are a simple, patient-friendly alternative to conventional methods. These devices require a small amount of blood, are minimally invasive as the sample is obtained by finger prick, and do not need specialized personnel. Patients can even be trained to obtain the samples at their homes, and send them to the laboratory by mail, minimizing patients’ trips to the hospital.

However, before these microsampling methods can be used for TDM, more data is needed to establish the correlation between venous plasma concentrations and peripheral blood concentrations obtained by finger prick.

Some methods have been previously published for the determination of AEDs using DBS, most detecting 1 to 5 drugs [2–20], with few exceptions [21, 22]. For VAMS, only 5 methods reported the detection of AEDS [21, 23–26]. In addition, Velghe et al. [26] collected both DBS and VAMS samples, and compared results observed in the two sample devices for valproic acid (VPA), carbamazepine (CBZ), and phenobarbital (PHB).

In the present study, we describe a method for the analysis of 8 AEDs and 2 metabolites (10,11-Dihydro-10-hydroxy-carbamazepine (DHCB), oxcarbazepine (OX) metabolite; and carbamazepine-10,11-epoxide (CBZE), CBZ metabolite) in DBS and VAMS samples. The method was validated and applied to real samples from 80 patients under treatment with one or more of the AEDs included in the methodology. A first estimate of the possible correlation between the matrices was performed by comparing concentrations in DBS and VAMS with those observed in paired plasma and whole blood samples.

## Materials and methods

### Chemicals and reagents

Reference standards for CBZE, levetiracetam-d_6_, PHB, phenobarbital-d_5_ in methanol, and 10,11-Dihydro-10-hydroxy-­carbamazepine ^13^C_6_, lacosamide (LA), lacosamide ^13^Cd_3_, and oxcarbazepine ^13^C_6_ in acetonitrile were purchased from Sigma-Aldrich (Merck KGaA, Darmstadt, Alemania).

VPA, valproic acid-d_4_, CBZ, carbamazepine-d_10_, lamotrigine (LMT), lamotrigine-d_3_, levetiracetam (LEV), OX, phenytoin (PHT), phenytoin-d_10_, and DHCB in solid form were purchased from Labclinics (Barcelona, Spain).

LC-MS grade acetonitrile (ACN) and methanol (MeOH) were purchased from Fisher Chemical (Loughborough, UK), and reagent grade acetic acid (AA) 98-100% was from Scharlab (Sentmenat, Spain). Water was purified with a Milli-Q water system (Millipore, Le-Mont-sur-Lausanne, Switzerland).

Mitra^TM^ VAMS devices (20 µL) were obtained from Neoteryx (Torrance, CA, USA), and Whatman™ 903^®^ Protein Saver cards from Cytiva (USA).

### Preparation of working solutions

AEDs were classified in four different groups (A to D) according to their therapeutic range. Initial working solutions in methanol were prepared at 20.25 mg/mL for group A (VPA) and group B (DHCB, LEV, PHT), 8.1 mg/mL for group C (LA, LMT, CBZ, PB), and 4.045 mg/mL for group D (CBZE, OX).

From the initial solutions, 400 µL (group A) or 100 µL (groups B, C, D) were diluted with MeOH to obtain 1 mL of the working solution used to prepare the highest calibrator, which was further diluted to get working solutions for the remaining calibrators of the curve.

For all the analytes, the corresponding labeled internal standard (IS) was employed. A solution containing all IS at different concentrations was prepared by dilution of the individual standards at 0.1 mg/mL. The final solution contains groups A and B at 16 µg/mL, group C at 6.4 µg/mL, and group D at 3.2 µg/mL in methanol.

### Preparation of calibrators and quality controls

For each calibrator and QC sample, 25 µL of the appropriate working solution was added to 950 µL of blank blood samples donated by the regional blood transfusion center. After agitation for 30 minutes, VAMS and DBS were prepared. For VAMS, the Mitra^TM^ device was put in contact with the blood and separated 2 seconds after it was filled, adsorbing 20 µL. For DBS, 35 µL of blood was deposited on the filter paper. Both devices were dried for at least 2 hours at room temperature.

Calibrator and QC concentrations for each analyte are detailed in Table 1.

**Table 1.** Calibrations and quality controls (QC) concentrations (µg/mL) for the antiepileptic drugs included in the methodology.

Group^a^	Cal 1	Cal 2	Cal 3	Cal 4	Cal 5	Cal 6	Cal 7
A	10	20	30	45	67.5	101.25	202.5
B	2.5	5	7.5	11.25	16.875	25.3125	50.625
C	1	2	3	4.5	6.75	10.125	20.25
D	0.5	1	1.5	2.25	3.375	5.0625	10.125
	LOQ	Low QC		Medium QC		High QC	

a Group A: valproic acid; Group B: 10,11-dihydro-10-hydroxy-carbamazepine, levetiracetam, phenytoin; Group C: lacosamide, lamotrigine, carbamazepine, phenobarbital; Group D: oxcarbazepine, carbamazepine-10,11-epoxide

### Sample clean-up

The center of the DBS was cut using a 6 mm punch, and the head of the Mitra™ was separated, applying the same clean-up protocol for both microsampling devices. Samples were introduced in a 2 mL Eppendorf tube. Then, 40 µL milliQ water, 20 µL IS and 140 µL MeOH were added, vortexing after each addition. The samples were incubated for 30 minutes in a hori­zontal shaker at room temperature, centrifuged 5 minutes at 14 500 rpm, and the supernatant was evaporated. Samples were reconstituted in 50 µL of mobile phase A: B (60:40, v/v) (A: ammonium acetate 5 mM in water, pH 4.5; B: ammonium acetate 5 mM in ACN: water (95:5, v: v), pH 4.5), centrifugated 5 minutes at 14 500 rpm and injected in the LC-MS/MS.

### LC-MS/MS

Samples were analyzed with a UPLC-MS/MS system, consisting of an Acquity UPLC^®^ H-Class chromatograph (Waters Corp. Milford, MA, USA) coupled to a Xevo^®^ TQ-XS mass spectrometer (Waters Corp.).

For the chromatographic separation, an ACQUITY UPLC^®^ CORTECS T3 (2.1 × 100 mm, 1.6 μm) column with a CORTECS T3 precolumn (2.1 x 5 mm, 1.6 μm) was selected. A different gradient was applied for the analytes detected using electrospray (ESI) in negative (VPA, PB, and PHT) or positive modes (remaining analytes), using the mobile phase described in section sample clean-up. The gradient for the ESI-method started at 20% B, increased to 60% in one min, to 98% until min 1.5, maintained until min 2.5, and decreased to 20% at min 2.6, with a total time of 4 min. The ESI+ method started at 10% B, increased to 27% at 0.22 min, and held until min 0.5, then increased to 31% at min 2.22, to 98% at min 3, and returned to initial conditions at min 3.9, with a total time of 6 min. For both methods, the flow rate was 0.5 mL/min, and column temperature was 45°C.

The MS was operated with the following parameters: capillary voltage, 3 kV (ESI+), 4 kV (ESI-); source block temperature, 150°C; desolvation gas (nitrogen) temperature, 250°C and desolvation and cone gas (nitrogen) flow rate, 600 and 150 L/h, respectively. Argon was employed to promote analyte fragmentation in the collision cell (flow rate, 0.15 mL/min). Data acquisition was performed with the MassLynx software (version 4.2 SNC 1035), and TargetLynx software was employed for data processing (Waters Corp.).

Two µL were injected for the analysis of the analytes detected in ESI+ mode and 5 µL for those detected in ESI- mode. Table 2 contains the MRM transitions and MS conditions for the compounds studied.

**Table 2.** MRM transitions, retention time, and mass spectrometric parameters for each analyte detected by electrospray in positive (ESI+) and in negative (ESI–) modes.^a^

Method I (ESI-)				
Compound	MRM transition	CV (V)	CE (eV)	Rt (min)
PHT	**251.0 > 102.0**	6	20	1.85
	251.0 > 77.0	6	26	1.85
PHT-d_10_	261.1 > 106.0	32	22	1.84
PHB	**231.1 > 42.0**	30	15	1.57
	231.1 > 188.1	30	10	1.57
PHB-d_5_	236.1 > 193.5	30	10	1.56
VPA	**143.1 > 143.1**	5	6	2.12
	143.1 > 143.1	35	6	2.12
VPA-d_4_	147.01 > 147.01	25	5	2.12

Method II (ESI+)				
Compound	MRM transition	CV (V)	CE (eV)	Rt (min)
CBZ	**237.2 > 179.0**	40	32	3.43
	237.2 > 165.1	40	36	3.43
CBZ-d_10_	247.0 > 204.1	2	31	3.40
LMT	**256.0 > 144.9**	2	32	1.58
	256.0 > 158.96	2	26	1.58
LMT-d_3_	261.1 > 191.3	2	20	1.56
LA	251.0 > 91.1	20	28	1.68
	**251.0 > 108.1**	20	28	1.68
LA-^13^Cd_6_	255.1 > 120.1	40	30	1.66
LEV	**171.2 > 126.1**	15	13	1.07
	171.2 > 98.1	15	22	1.07
LEV-d_6_	177.25 > 132.2	15	13	1.04
OX	**253.2 > 208.0**	50	27	2.64
	253.2 > 180.0	50	28	2.64
OX-^13^C_6_	259.2 > 171.2	30	20	2.64
CBZE	**253.2 > 180.1**	16	22	2.41
	253.2 > 167.1	16	32	2.41
CBZE-^13^C_6_	259.2 > 186.1	2	30	2.41
DHCB	**255.2 > 237.2**	18	8	1.99
	255.2 > 194.2	18	20	1.99
DHCB-^13^C_6_	261.1 > 243.2	18	8	1.99

a The MRM transition used for quantification is set in bold format. CBZ: carbamazepine; CBZE: carbamazepine-10,11-epoxide; CE: collision energy; CV: cone voltage; DHCB: 10,11-dihydro-10-hydroxy-carbamazepine; LA: lacosamide; LEV: levetiracetam; LMT: lamotrigine; OX: oxcarbazepine; PHB: phenobarbital; Rt: retention time; VPA: valproic acid.

### Analytical validation

The method was validated in DBS and VAMS according to the recommendations of the European Medicines Agency (EMA) guidelines [27], including linearity, selectivity, accuracy, precision, carryover, matrix effect, recovery, process efficiency, and stability. In addition, specific parameters for DBS validation were also studied as recommended by the International Association for Therapeutic Drug Monitoring and Clinical Toxicology Guideline [28], including the volume effect, hematocrit (Hct) effect, and volcano effect.

Details for the evaluation of these parameters and acceptance criteria are summarized in Table 3.

**Table 3.** Validation parameters, evaluation procedure, and acceptance criterio.

Validation parameter	Evaluation	Acceptance criteria
Linearity	6 calibrators + zero + blank 5 curves in 5 different days	*r* ^2^ ≥ 0.99 Calibrators residuals ±15% (±20% at LLOQ)
Selectivity	Endogenous interferences: 6 different blank matrices Exogenous interferences: Blank matrices (*n* = 2) fortified with common pharmaceuticals^a^ at 12.5 µg/mL	≤20% of LLOQ nominal concentration (analyte)
Accuracy and precision	At 4 levels: LLOQ, low, medium, and high QC concentrations (*n* = 15 each)	Accuracy: 85–115% of nominal concentration Precision: %CV ≤15%
Carryover	Analysis of blank samples after the highest calibrator	≤ 20% of LLOQ nominal concentration (analyte) ≤ 5% (IS)
Matrix effect	Comparing APA at low and high QC concentrations (*n* = 6) when the analytes were fortified in reconstitution solvent with APA in blank samples fortified after extraction (*n* = 6 donors) 2 additional blank samples at Hct 0.2 and 0.6 were prepared from a donor’s WB with 0.4 Hct.	%CV IS-MF ≤15%
Recovery	Comparing APA at low and high QC (*n* = 6) blank samples fortified before extraction (*n* =6 each condition) with APA in blank samples fortified after extraction (*n* = 6 each condition). For VAMS, blank samples at Hct 0.4 were used For DBS, blank samples at different Hct (0.2, 0.4 and 0.6, *n* = 5 each) were employed	Hct effect on recovery: One-way ANOVA with Bonferroni post hoc analysis (*P* ≤ .05)
Stability	At low and high QC concentrations by comparing concentrations in fresh samples (*n* = 3) with those obtained in: (A) Extracts stored 72 h in autosampler at 6°C (B) Samples stored 15 days at room temperature before extraction	Stability samples should quantify within ±15% of freshly prepared QC samples
DBS specific parameters		
Homogeneity (Volcano effect)	At low and high QC concentrations and 3 Hct levels (0.2, 0.4, and 0.6) using 60 µL blood by comparing concentrations in the central punch with those at peripheral punches (*n* = 3)	Paired *t* test (*P* ≤ .05) Back calculated “peripheral” values deviation ≤15% of “central” values
Volume effect	At low and high QC concentrations and 3 Hct levels by comparing concentrations when applying 35 µL blood to those obtained using different volumes (20 µL, 25 µL, 45 µL and 60 µL)	One-way ANOVA with Bonferroni post hoc analysis (*P* ≤ .05) Back calculated values deviate ≤15% of 35 µL samples

APA: average peak area; Hct: hematocrit; WB: whole blood; IS-MF: internal standard normalized matrix factor.

a Exogenous interferences included mycophenolic acid, cyclosporine A, tacrolimus, everolimus, sirolimus, bromadiolone y warfarin, acetylsalicylic acid, metamizole, piroxicam, metformin, citalopram, trazodone, fluoxetine, sertraline, venlafaxine, gemcitabine, irinotecan, dacarbazine, pemetrexed, vinorelbine, doxorubicin, docetaxel, epirubicin, methotrexate, cyclophosphamide, etoposide, paclitaxel, clozapine, haloperidol, levomepromazine, olanzapine and quetiapine.

### Application to real samples

Between March 2023 and June 2024, 80 real samples were collected from children (1-17 y.o.; *n* = 71) and adult (24-66 y.o.; *n* = 24) patients under treatment with one (*n* = 56) or more (*n* = 24) of the studied AEDs. Forty-five of the participants were male, and 35 were female, with Hcts in the range 0.332–0.508 (other participant data is included in Supplementary Table S1; see online supplementary material for a color version of Table S1). One tube of venous blood, two DBS samples, and one VAMS sample were obtained during routine checkup by medi­cal professionals. Samples were sent to our laboratory and stored at −20°C until analysis. Time between collection and analysis ranged between 1 and 12 months.

This study has the approval of the Ethics Committee, and procedures were in accordance with the tenets of the Declaration of Helsinki. All volunteers were informed both verbally and in writing about the research and signed a written consent before sampling.

## Results

### Method development and analytical validation

Chromatographic conditions allowed elution of all the analytes within 3.5 min in ESI+ mode, and 2.2 min in ESI- mode. For the detection, the most abundant transition for each analyte was employed for quantification, and a second transition was monitored for qualitative purposes. VPA was not fragmented, as observed by previous authors [9, 25], and a pseudotransition (143.1 > 143.1) was used as a surrogate.

Linearity was verified by least squares regression using 1/x weighing factor, in a range encompassing each AED therapeutic range (Table 1), and *r*^2^ for all compounds was >0.99 in VAMS and DBS samples.

No endogenous or exogenous interferences were detected in any case.

Accuracy and imprecision acceptance criteria were met for all analytes.

For all QCs, accuracy was 95.7–102.1% for VAMS and 93.7–103.6% for DBS. Intra-day, inter-day, and total imprecision (%CV) were <11%, <13.3%, and <11.7%, respectively, for VAMS, and <13.6, <10.2%, and <13.6%, respectively, for DBS. Specific data for all the analytes are shown in Supplementary Table S2 (see online supplementary material for a color version of Table S2).

Carryover was determined by analyzing a blank sample after injection of the highest calibrator. No carryover was detected for any analyte.

Matrix effect for each analyte was normalized taking into consideration the effect for its IS (IS normalized matrix factor, IS-MF). IS-MF range between 0.91—1.11 for VAMS and 0.89—1.07 for DBS, and the %CV was <15% in all cases. Detailed data is shown in Supplementary Table S3 (see online supplementary material for a color version of Table S3).

Recovery in VAMS was 88.4%–97.1% for the different analytes. Recovery for DBS was much lower, ranging from 6.2% to 44.3% for Hct 0.2, 7.9%–46.6% for Hct 0.4, and 18.6% − 62.5% for Hct 0.6. Detailed data is shown in Supplementary Table S4 (see online supplementary material for a color version of Table S4).

Regarding the stability studies, %loss in VAMS samples stored at room temperature for 15 days ranged between −10.3% and 4%, and in DBS samples between −6.8% and 4.7%, except for OX, which was unstable (−61.8% and −33.3%) under these conditions. For autosampler stability, %loss was −9.4% to 4.2% for VAMS samples, and −3.2 to 7.8% for DBS, except for LA (39% to 40.6%) and OX (−23.5% to −24.7%). These results are shown in Supplementary Table S3 (see online supplementary material for a color version of Table S3).

For DBS samples, the volume effect, volcano effect, and the Hct effect were also assessed. No differences were observed when using different blood volumes as back calculated values deviated ≤15% from the reference volume (35 μL) regardless of the Hct. Detailed data for the volume effect study is available as Supplementary Table S5 (see online supplementary material for a color version of Table S5).

DBS homogeneity was verified for all the analytes as back calculated peripheral values deviated ≤15% from the central values, except for PHT at high QC concentration and Hct 0.2. Detailed results are available at Supplementary Table S6 (see online supplementary material for a color version of Table S6).

No Hct impact was observed on recovery after application of one-way ANOVA with Bonferroni *post hoc* analysis (*P* < .05) at Hcts 0.2 and 0.4, except for LEV and OX at high QC, but a difference was observed between Hcts 0.4 and 0.6 for all compounds (Supplementary Table S4; see online supplementary material for a color version of Table S4).

### Application to real samples

Results in venous blood, DBS, and VAMS samples were compared to those obtained by routine plasma analysis at the hospital to obtain a preliminary overlook of how concentrations compare in the different matrices.

In Spain, VPA and LEV are the most prescribed AEDs, thus, most of our participants were prescribed these drugs. Specifically, 39 and 27 participants were under treatment with VPA and LEV, respectively. These patients tested positive in all matrices with two exceptions. One patient had VPA concentrations lower than the lower limit of quantification (LLOQ) in all samples, and another had LEV concentrations <LLOQ in plasma, and undetectable in the other 3 samples. Less than 15 participants were under treatment with OX (*n* = 5), LMT [12], LA (*n* = 7), CBZ [7], and/or PHB (*n* = 3). For all the AEDS, the parent drug was identified in the corresponding patients, except for OX (only detected in 5 out of the 13 treated patients at concentrations close to the LLOQ in blood and DBS samples), for which the metabolite DHCB was the main analyte detected (*n* = 13) due to its quick metabolization [29]. CBZ’s metabolite CBZE was detected in 5 of 7 patients, in some cases only in venous blood and DBS. We did not receive any samples from patients prescribed with PHT.

**Figure 1.**
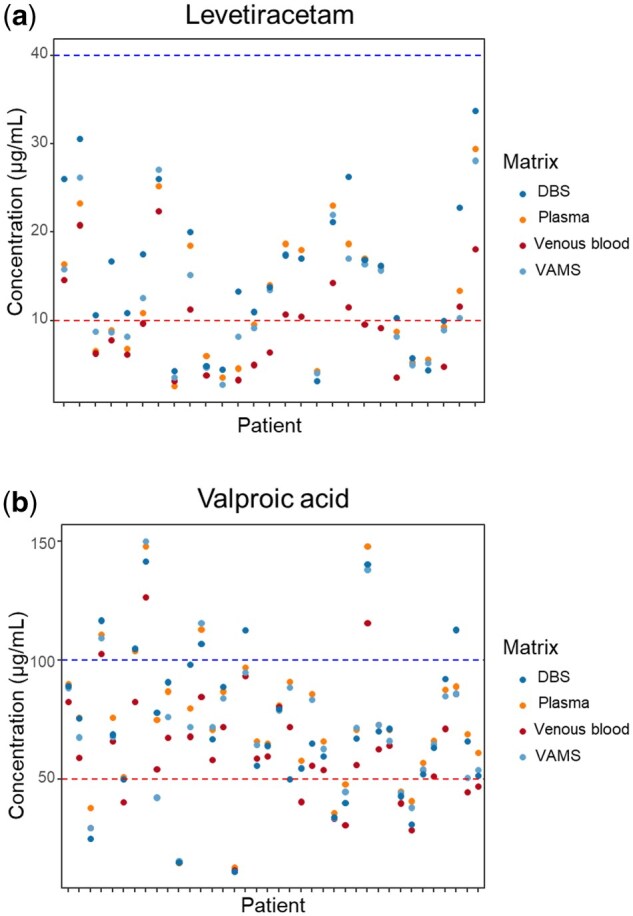
Concentrations found in levetiracetam (LEV) positive samples (a) and in valproic acid (VPA) positive samples (b). The X-axis represents individual patients (patient number not shown). The striped lines in the Y-axis mark the lower and upper limits of the therapeutic ranges.

For most AEDS, concentrations were within their respective therapeutic ranges (Table 4). For LEV, 13 plasma samples (48.1%) showed concentrations below the therapeutic range, close to our LLOQ. For VPA, 7 plasma samples (18.4%) were below the therapeutic range, and 5 (13.2%) were over the therapeutic range. Figure 1 shows concentrations found in positive LEV (1A) and VPA (1B) samples.

**Table 4.** Positive cases and concentration ranges (µg/mL) in plasma, venous blood, VAMS, and DBS samples from patients under AEDs treatment.

Analyte	Positive samples (*n*)	Concentrations range (µg/mL)				Reference TR (µg/mL)
		Plasma	Venous blood	VAMS	DBS	
VPA	39	13.0–148.0	11.9–126.6	11.2–150.2	11.3–141.6	50–100
LEV	27	2.6–29.4	2.5–22.4	2.8–28.1	3.2–33.7	10–40
DHCB	13	9.3–33.5	3.9–32.0	2.6–32.9	4.0–41.1	3–35
LMT	12	2.1–11.7	1.0–11.5	1.3–11.2	1.1–16.2	3–14
LA	7	4.1–8.8	1.3–8.5	2.1–9.1	1.9–11.4	1–10
CBZ	7	2.2–10	1.8–11.9	1.9–8.1	2.0–9.5	4–10
CBZE	5	N/A	1.1–2.7	0.9–1.2	1.2–2.2	–
OX	5	N/A	0.5–1.1	<LOQ	0.5–1.7	–
PHB	3	21.0–41.0	36.4–73.1	16.2–43.0	13.0–38.2	10–40

CBZ: carbamazepine; CBZE: carbamazepine-10,11-epoxide; DHCB: 10,11-dihydro-10-hydroxy-carbamazepine; LA: lacosamide; LEV: levetiracetam; LMT: lamotrigine; OX: oxcarbazepine; PHB: phenobarbital; VPA: valproic acid; TR: therapeutic range.

Blood/plasma ratios were calculated for the positive samples obtained from the same individuals, using plasma concentrations reported by the hospital reference laboratory and venous blood concentrations obtained in our laboratory. Ratios were also calculated for capillary blood using paired VAMS and DBS concentrations. Finally, VAMS/DBS ratios were calculated to compare the two sampling techniques. Mean ratios and their %CV between patients are shown in Supplementary Table S7 (see online supplementary material for a color version of Table S7). The venous blood/plasma ratio was close to 1 for DHCB, LMT, and CBZ; <1 for VPA, LEV, and LA; and >1 for PHB. VAMS/plasma and DBS/plasma ratios were closer to 1, except for PHB (DBS/plasma ratio = 0.78; %CV = 20.2%). Specifically, VAMS concentrations tend to be lower than plasma concentrations in most cases, and DBS concentrations are slightly higher. VAMS/DBS ratios also varied between drugs, with similar concentrations in both microsamples for VPA and LA, lower concentrations in VAMS for LEV, LMT, DHCB, and LA, and higher VAMS concentrations for PHB. In addition, for VPA and LEV, the most frequent AEDS detected, a simple statistical comparison between the different matrices was performed. Supplementary Figure S1 (see online supplementary material for a color version of Figure S1) shows boxplots of LEV (1A) and VPA (1B) concentrations in the different matrices. When comparing mean concentrations with a Wilcoxon signed-rank test, no significant differences were observed between concentrations in the different matrices, except for venous blood vs. plasma concentrations for VPA, and for venous blood vs. DBS concentrations for LEV.

## Discussion

We present a method for the quantification of 8 antiepileptic drugs and 2 metabolites in both VAMS and DBS samples. To date, most published methods for blood microsampling are centered in one to five AEDs [2–20, 23, 24, 26], with only two exceptions. D’Urso et al. [21] published a method for the detection of 14 AEDs and 2 metabolites in VAMS, and Moller et al. [22] presented a method for the detection of 22 AEDs and 5 metabolites in DBS. However, Moller’s method did not include drugs that can be ionized only in negative mode, such as VPA (one of the most common AEDs used in the clinical practice), PHB or PHT, and d’Urso’s method did not include VPA. Our method includes a two-injection process, allowing the detection of drugs in both ionization modes in a short time, with total run times of 4 min and 6 min for the negative and positive ionization methods, respectively. Furthermore, the method was validated and can be applied to paired DBS and VAMS samples to more easily compare results in both devices.

The method was satisfactorily validated for both VAMS and DBS samples. LOQs (Table 1) were low enough to include the therapeutic range of the respective drugs, and in most cases, even lower to allow the detection of subclinical concentrations.

An instability problem after 72 h in the autosampler was found for LA, specifically due to the instability of the IS lacosamide-^13^Cd_3_, so reanalysis of samples after 48 h was avoided. This is not expected to affect the applicability of the method, as in the event of instrument failure, samples can usually be injected within the following 24 h. OX was also unstable in DBS after 15 days at room temperature and after 72 h in the autosampler; however, OX is barely detected in blood after ingestion, as reflected in our results, and it is not used for TDM in clinical practice.

Matrix effects were similar in VAMS and DBS samples, and negligible when normalized with the IS. Recovery, however, was much lower for DBS samples than for VAMS samples. A lower recovery for DBS has also been reported for some of the drugs by Velghe et al. [19]. Nevertheless, sensitivity was not an issue, and concentrations detected in real samples also show a good correlation when comparing DBS and VAMS concentrations.

For the DBS-specific validation, effect of the blood volume was not significant in the range of 20-45 µL, although one-way ANOVA analysis showed a significant statistical difference for Hct 0.2 and high QC using 60 µL for some of the drugs (Supplementary Table S5; see online supplementary material for a color version of Table S5). Homogeneity of the spots showed ≤15% difference between central and peripheral spots for all drugs except for PHT at low QC and Hct 0.2, and t test showed no significant difference except for CBZ at low QC and Hct 0.2. To avoid homogeneity problems, punches were taken from the center of the spots for all samples.

For DBS samples, an effect of the Hct on the recovery was observed, with an increase in the recovery with higher Hct, and a statistically significant difference was found between the reference Hct 0.4 and the highest Hct (0.6) for all compounds. This was not an issue for the study since none of our participants had an Hct higher than 0.5.

The method was applied to paired VAMS and DBS samples from patients under AEDs treatment, being able to detect the corresponding drug in all cases where hospital concentrations in plasma were also positive. CBZ metabolite CBZE was only detected in some patients. On the contrary, OX metabolite DHCB was the analyte detected in all patients under OX treatment, with just 50% of the cases positive for OX. The present method is the first to include DHCB in DBS and VAMS samples which, according to our results, is essential to monitor OX. Furthermore, despite not having enough samples for a full statistical evaluation, VAMS/plasma and DBS/plasma ratios show a good correlation between these microsamples and plasma concentrations.

Most of the patients were being treated with LEV and VPA, so a more in-depth study of the concentrations in the different matrices was carried out for these compounds. Regarding the concentrations observed in the real samples, 18.4% (VPA) and 50% (LEV) of cases were below therapeutic concentrations, and 13.2% over VPA’s therapeutic limit. Moreover, in two cases, patients under treatment with LEV and VPA had concentrations below our LLOQ, and the analytes were also undetected in plasma samples. When looking at the venous blood/plasma ratios, we observe concentrations higher in plasma than blood for VPA (mean ratio 0.83), meaning VPA is mainly present in the plasma fraction of the blood. This was also observed in DBS and VAMS, although these samples presented ratios closer to 1 in our participants (mean 0.95 and 0.94, respectively). Linder et al. [10, 11] also found higher concentrations in plasma than blood (in DBS) for VPA. Other authors [14, 15, 25] also reported higher VPA concentrations in plasma than whole blood, DBS or VAMS samples. Furthermore, our VAMS/DBS ratio was 1.01, showing a good agreement between both microsampling techniques.

For LEV, our venous blood/plasma ratio was even lower (mean ratio 0.71), showing also higher concentrations of this compound in plasma. Besten-Bertholee et al. [2] found higher concentrations in plasma than in DBS samples, but Linder et al. [11] found similar concentrations when comparing DBS and plasma concentrations. Our VAMS/plasma ratio was close to 1 (1.04), similar to that found by Linder et al. [11], but our DBS/plasma ratio was 1.33, indicating higher concentrations in DBS than plasma, the opposite to what Besten-Bertholee et al. [2] found. A VAMS/DBS ratio of 0.82 also shows higher concentrations in DBS than plasma in our patients.

The usefulness of the method for the detection of antiepileptic drugs was demonstrated with its application to real samples from patients under AEDs treatment. Although the described preliminary results show a good agreement between concentrations in microsampling devices and plasma, analysis of more real samples should be performed to obtain a statistically sound comparison of the concentrations in the different matrices.

## Conclusion

A method for the analysis of 8 antiepileptic drugs and 2 metabolites was developed and validated for DBS and VAMS samples. The method was applied to paired blood, VAMS and DBS samples from patients, finding good concordance between plasma and DBS and VAMS samples for VPA and LEV. In the future, additional samples will be analyzed to complete the clinical validation in VAMS and DBS samples, with the aim of applying the method for therapeutic drug monitoring of these drugs.

## Supplementary Material

bkaf073_Supplementary_Data

## Data Availability

The data underlying this article will be shared on reasonable request to the corresponding author.

## Other information

The following information was extracted from section “2.7. Application to real samples” to maintain the blinded review:

“Real samples were obtained from patients from the University Hospitals of Santiago de Compostela (CHUS), Vigo (CHUVI), and Ferrol (CHUF), in the northwest of Spain.”

This sentence could be included again within the section if accepted.
