# Supplementary material for: Development and application of an LC-MS/MS method for 8 antiepileptic drugs and 2 metabolites using microsampling techniques (DBS and VAMS)
Source: J Anal Toxicol. 2025 Jul 16;50(1):bkaf073. doi: 10.1093/jat/bkaf073 (PMC13168808; doi:10.1093/jat/bkaf073)
Supplement: bkaf073_Supplementary_Data [file bkaf073_supplementary_data.pdf]

Table S1. Participants demographic and anthropometric data

**Number of participants**	80
**Age range (mean ± SD; median)**	1–66 (14.4 ± 14; 11)
**Height range (mean ± SD; median) (cm)**	75–189 (140.8 ± 26.7; 143.5)
**Weight range (mean ± SD; median) (kg)**	9.5–116 (46 ± 25; 38.3)
**Hematocrit range (mean ± SD; median) (%)**	33.2–50.8 (40.5 ± 3.7; 40.1)

Table S2. Linearity, LOQ, accuracy and imprecision results in DBS and VAMS samples

DBS	R^2	LOQ	Accuracy	Intra-day imprecision	Inter-day imprecision	Total imprecision	
**Levetiracetam**	0.9966	2.5	98.4 (5.4)	13.6	0.0	13.6	Low QC
			93.7 (4.9)	4.9	2.2	5.4	Medium QC
			97.4 (4.6)	3.8	0.0	3.8	High QC
**Lacosamide**	0.9958	1	98.2 (7.4)	6.8	3.6	7.7	Low QC
			95.6 (4.7)	7.0	1.4	7.2	Medium QC
			99.7 (6.6)	5.7	0.0	5.7	High QC
**Lamotrigine**	0.9946	1	97.9 (7.5)	7.4	4.6	8.7	Low QC
			97.5 (6.8)	5.4	6.6	8.5	Medium QC
			95.4 (5.4)	4.3	2.8	5.1	High QC
**10,11-Dihydro-10-hydroxy-carbamazepine**	0.9971	2.5	96.5 (6.1)	4.7	2.1	5.2	Low QC
			97.0 (6.9)	4.1	8.2	9.1	Medium QC
			96.4 (6.6)	3.9	1.9	4.3	High QC
**Carbamazepine-10,11-epoxide**	0.9967	0.5	96.8 (7.7)	4.4	5.4	6.9	Low QC
			98.0 (7.0)	4.2	7.5	8.6	Medium QC
			97.5 (6.9)	4.6	0.6	4.7	High QC
**Oxcarbazepine**	0.9955	0.5	99.5 (7.8)	4.5	0.0	4.5	Low QC
			98.4 (6.6)	5.1	6.5	8.3	Medium QC
			97.9 (6.9)	4.7	1.4	4.9	High QC
**Carbamazepine**	0.9942	1	96.3 (7.3)	6.5	1.4	6.7	Low QC
			98.4 (8.0)	6.7	7.0	9.7	Medium QC
			97.5 (5.9)	4.7	0.0	4.7	High QC
**Valproic acid**	0.9969	10	97.3 (6.2)	5.0	1.7	5.3	Low QC
			103.6 (4.7)	5.2	0.0	5.2	Medium QC
			99.6 (3.6)	3.3	0.0	3.3	High QC
**Phenobarbital**	0.9979	1	95.8 (7.6)	7.6	0.0	7.6	Low QC
			98.8 (7.7)	3.3	10.2	10.7	Medium QC
			98.8 (9.8)	4.9	3.6	6.0	High QC
**Phenytoin**	0.9953	2.5	96.7 (7.2)	6.9	0.0	6.9	Low QC
			94.7 (4.8)	4.4	4.1	6.0	Medium QC
			93.7 (8.4)	5.5	2.7	6.1	High QC

VAMS	R^2	LOQ	Exactitude	Intra-day imprecision	Inter-day imprecision	Total imprecision	
**Levetiracetam**	0.997	2.5	101.5	5.7	0.0	5.7	Low QC
			97	1.8	0.0	1.8	Medium QC
			95.7	3.0	1.9	3.6	High QC
**Lacosamide**	0.9953	1	102.1	6.9	3.1	7.5	Low QC
			102.1	2.7	3.7	4.5	Medium QC
			100.1	4.4	5.7	7.2	High QC
**Lamotrigine**	0.9966	1	97.9	7.1	3.1	7.8	Low QC
			101.8	3.9	7.6	8.5	Medium QC
			98.4	1.9	1.5	2.5	High QC
**10,11-Dihydro-10-hydroxy-carbamazepine**	0.9993	2.5	101	7.2	0.0	7.2	Low QC
			97.4	1.5	1.7	2.3	Medium QC
			99.5	5.1	9.4	10.7	High QC
**Carbamazepine-10,11-epoxide**	0.9960	0.5	101.6	3.4	13.3	13.8	Low QC
			96.2	4.6	8.8	10.0	Medium QC
			97	4.5	6.0	7.5	High QC
**Oxcarbazepine**	0.9976	0.5	100.7	9.0	2.8	9.4	Low QC
			96.3	6.1	10.0	11.7	Medium QC
			97	2.6	3.7	4.5	High QC
**Carbamazepine**	0.9972	1	101.9	9.6	0.0	9.6	Low QC
			97.4	3.7	3.0	4.7	Medium QC
			98.2	1.9	3.5	4.0	High QC
**Valproic acid**	0.9963	10	101.8	5.3	0.0	5.3	Low QC
			98.9	2.5	0.0	2.5	Medium QC
			104.1	4.5	4.8	6.6	High QC
**Phenobarbital**	0.9989	2.5	99.2	11.0	0.0	11.0	Low QC
			96.4	3.9	6.5	7.6	Medium QC
			96.2	4.0	6.2	7.3	High QC
**Phenytoin**	0.997	1	99.4	6.7	4.5	8.1	Low QC
			101.8	2.7	2.1	3.4	Medium QC
			100.4	6.8	0.0	6.8	High QC

Table S3. Matrix effect and stability in autosampler and stability after 15 days at room temperature (RT) in DBS and VAMS samples

DBS		IS-MF (%CV)	Stability autosampler (% diff)	Stability 15 days at RT (% diff)
**Levetiracetam**	Low QC	1.02 (4.32)	2	1.8
	High QC	0.99 (2.61)	0.3	2.5
**Lacosamide**	Low QC	1.01 (4.9)	39.0	–0.4
	High QC	0.98 (2.4)	40.6	1.4
**Lamotrigine**	Low QC	1.04 (6.4)	–1.0	0.0
	High QC	1.00 (3.27)	0.2	1.4
**10,11-Dihydro-10-hydroxy-carbamazepine**	Low QC	1.01 (5.0)	5.3	–1.2
	High QC	0.99 (2.03)	7.8	3.1
**Carbamazepine-10,11-epoxide**	Low QC	1.00 (5.78)	–2.0	–2.3
	High QC	0.99 (2.36)	–0.9	4.7
**Oxcarbazepine**	Low QC	0.99 (5.43)	–23.5	–33.3
	High QC	0.98 (2.85)	–24.7	–61.8
**Carbamazepine**	Low QC	0.98 (4.33)	–1.0	0.9
	High QC	0.99 (1.19)	–3.2	3.3
**Valproic acid**	Low QC	1.00 (5.93)	–1.4	–0.5
	High QC	0.89 (10.23)	–1.6	–6.1
**Phenobarbital**	Low QC	0.99 (9.19)	4.1	2.7
	High QC	1.07 (4.52)	2.2	4.3
**Phenytoin**	Low QC	0.96 (10.19)	3.8	–6.8
	High QC	1.03 (5.07)	1.7	0.2
**VAMS**		**IS-MF (%CV)**	**Stability autosampler (% diff)**	**Stability 15 days at RT (% diff)**
**Levetiracetam**	Low QC	1 (6.25)	4.2	–6.3
	High QC	0.96 (4.20)	4.2	–5.6
**Lacosamide**	Low QC	0.98 (5.12)	–8.1	–10.3
	High QC	0.95 (7.20)	–0.2	–0.1
**Lamotrigine**	Low QC	0.98 (14.24)	–9.4	–7.3
	High QC	0.95 (7.34)	–1.5	–3.1
**10,11-Dihydro-10-hydroxy-carbamazepine**	Low QC	0.95 (5.9)	–2.6	–9.9
	High QC	0.97 (5.96)	–3.9	–3.6
**Carbamazepine-10.11-epoxide**	Low QC	0.91 (7.03)	2.1	–10
	High QC	0.95 (9.12)	–1.4	–5.4
**Oxcarbazepine**	Low QC	1 (6.91)	3.5	–10.3

	High QC	0.95 (7.06)	3.6	−5.5
**Carbamazepine**	Low QC	0.96 (11.37)	−8.9	−5.4
	High QC	0.95 (3.3)	−0.7	−2.7
**Valproic acid**	Low QC	0.95 (5.51)	−3	2.3
	High QC	1 (5.72)	−3.8	3.3
**Phenobarbital**	Low QC	1.03 (6.52)	−9.2	4
	High QC	0.98 (5.81)	−3.9	2.9
**Phenytoin**	Low QC	1.11 (11.62)	3.4	−6.4
	High QC	0.98 (10.35)	1.1	−2.7
IS-MF: Internal standard-normalized matrix factor				

Table S4. Recovery in DBS and VAMS samples

**DBS**		Recovery (%)				Recovery (%)		
		Hct 0.2	Hct 0.4	Hct 0.6		Hct 0.2	Hct 0.4	Hct 0.6
**Levetiracetam**	Low QC	26.3	30.9	39.5*	**Levetiracetam-d_6**	65.4	65.2	64.8
	High QC	35.7*	41.6	55.6*		89.1	86.2	79.0
**Lacosamide**	Low QC	30.4	35.0	43.2*	**Lacosamide-$^{13}Cd_6$**	70.5	70.6	67.5
	High QC	39.0	43.5	60.4*		90.8	83.5	80.2
**Lamotrigine**	Low QC	44.3	44.8	51.3*	**Lamotrigine-d_3**	98.0	89.2	79.9
	High QC	43.4	46.6	61.6*		97.5	92.6	85.7
**10,11-Dihydro-10-hydroxy-carbamazepine**	Low QC	29.1	33.8	42.1*	**10,11-Dihydro-10-hydroxy-carbamazepine-$^{13}C_6$**	70.8	71.7	69.8
	High QC	36.8	41.0	57.4*		94.8	86.8	84.0
**Carbamazepine-10,11-epoxide**	Low QC	26.7	31.8	40.2*	**Carbamazepine-10,11-epoxide-$^{13}C_6$**	65.9	67.6	65.1
	High QC	34.7	39.2	55.4*		89.1	83.0	79.0
**Oxcarbazepine**	Low QC	14.6	19.8	24.1*	**Oxcarbazepine-$^{13}C_6$**	46.5	56.2	52.5
	High QC	18.4*	23.5	35.3*		64.9	68.1	68.0
**Carbamazepine**	Low QC	27.8	31.6	39.6*	**Carbamazepine-d_{10}**	68.7	67.9	66.9
	High QC	42.7	46.3	62.5*		80.0	74.7	75.5
**Valproic acid**	Low QC	6.2	7.9	18.6*	**Valproic acid-d_4**	16.5	16.6	29.4
	High QC	14.5	15.7	26.9*		27.4	24.1	28.9
**Phenobarbital**	Low QC	24.3	26.2	35.4*	**Phenobarbital-d_5**	54.8	58.2	60.0
	High QC	26.6	30.3	46.6*		68.7	67.6	67.2
**Phenytoin**	Low QC	25.1	30.1	40.6*	**Phenytoin-d_{10}**	64.8	68.4	77.5
	High QC	27.4	29.9	48.9*		70.7	66.8	69.5

**VAMS**		Recovery (%)		Recovery (%)
		Hct 0.4		Hct 0.4
**Levetiracetam**	Low QC	88.4	**Levetiracetam-d_6**	89.8
	High QC	89.2		91.1
**Lacosamide**	Low QC	88.7	**Lacosamide-$^{13}Cd_6$**	88.9
	High QC	88.9		90.3
**Lamotrigine**	Low QC	93.1	**Lamotrigine-d_3**	90.1
	High QC	95.2		91.1

**10,11-Dihydro-10-hydroxy-carbamazepine**	Low QC	90.1	**10,11-Dihydro-10-hydroxy-carbamazepine-$^{13}C_6$**	92.2
	High QC	94.5		95.7
**Carbamazepine-10.11-epoxide**	Low QC	95.3	**Carbamazepine-10,11-epoxide-$^{13}C_6$**	96.9
	High QC	95.9		97.1
**Oxcarbazepine**	Low QC	94.8	**Oxcarbazepine-$^{13}C_6$**	96.2
	High QC	95.6		95.7
**Carbamazepine**	Low QC	96.9	**Carbamazepine-d_{10}**	92.1
	High QC	97		92.9
**Valproic acid**	Low QC	94.7	**Valproic acid-d_4**	92.2
	High QC	95.5		93.6
**Phenobarbital**	Low QC	96.7	**Phenobarbital-d_5**	93.2
	High QC	97		90.8
**Phenytoin**	Low QC	97.1	**Phenytoin-d_{10}**	96.2
	High QC	96.9		95.4
*Statistically significant different from the 0.4 Hct reference QCs (one-way ANOVA with Bonferroni post-hoc analysis, $p \leq 0.05$) (n = 5)				

Table S5. Volume effect study in DBS samples

		Hct 0.2				Hct 0.4				Hct 0.6			
		**20 µL**	**25 µL**	**45 µL**	**60 µL**	**20 µL**	**25 µL**	**45 µL**	**60 µL**	**20 µL**	**25 µL**	**45 µL**	**60 µL**
**Levetiracetam**	Low QC	−7.8 (4.4)	−3.2 (7.1)	0.6 (8.9)	9.1 (3.6)	−12.7 (11.4)	−0.5 (2.4)	−0.5 (11.5)	12.7 (13.6)	3.7 (13.9)	2.3 (6.2)	−1.4 (0.8)	9.2 (4.4)
	High QC	−3.5 (6.6)	−1.5 (2.6)	6.9 (2.8)	12.9* (1.7)	−1.8 (5.9)	−1.9 (6.3)	0.3 (5.5)	4.0 (9)	1.6 (4.3)	−4.2 (4.8)	−1.6 (1)	0.0 (8)
**Lacosamide**	Low QC	3.6 (5.3)	−1.8 (5.6)	−1.8 (5.6)	1.8 (8.2)	−3.1 (14.3)	4.6 (2.5)	−3.1 (12.6)	10.8 (14.4)	9.9 (13.3)	8.5 (9.8)	−4.2 (2.5)	11.3 (4.4)
	High QC	−2.6 (6)	−3.0 (2)	5.2 (1.9)	12.9* (2.4)	−2.9 (5)	−3.5 (8.2)	−1.2 (6.6)	2.7 (7.7)	2.9 (5.7)	−5.9 (6.3)	0.0 (0)	1.0 (9.4)
**Lamotrigine**	Low QC	6.2 (15.6)	−8.3 (3.9)	4.2 (9.2)	−4.2 (10)	−5.6 (11.8)	7.4 (7.9)	0.0 (11.1)	9.3 (11.7)	0.0 (14.2)	0.0 (10.2)	−8.2 (3.1)	3.3 (4.8)
	High QC	0.9 (6.1)	0.5 (0.8)	8.1 (3.5)	14.2* (1.9)	−1.7 (8.9)	−1.7 (8)	2.1 (7.1)	4.2 (10.1)	−6.8 (5.6)	−8.0 (6.4)	0.0 (2)	3.4 (8.3)
**10,11-Dihydro-10-hydroxy-carbamazepine**	Low QC	−6.1 (3.7)	−3.8 (3.6)	0.0 (7.9)	0.8 (6.9)	−7.6 (13.8)	5.1 (1.8)	−3.8 (11.6)	8.3 (13.4)	8.2 (16.4)	5.3 (8.3)	−5.8 (2.8)	6.4 (4.1)
	High QC	0.3 10	−1.4 1.6	7.7 (0)	13.1 (1.3)	−1.2 (7)	−3.7 (7.1)	0.1 (6.7)	1.8 (9.7)	−0.4 (4.5)	−5.7 (4.2)	−0.4 (0)	1.2 (7.1)
**Carbamazepine-10,11-epoxide**	Low QC	0.0 (6.9)	−4.0 (0)	0.0 (6.9)	12.0 (6.2)	−6.5 (11.9)	0.0 (5.6)	−6.5 (11.9)	6.5 (15.7)	9.1 (14.4)	9.1 (8.3)	−3.0 (5.4)	6.1 (4.9)
	High QC	−0.9 (7.9)	−5.2 (1.6)	5.2 (2.9)	12.2 (2.3)	−1.8 (5.6)	−3.0 (6)	−2.4 (5.4)	3.0 (10)	−2.0 (6.3)	−6.1 (7.5)	−2.0 (0)	−2.0 (6.3)
**Oxcarbazepine**	Low QC	−2.7 (8.3)	−2.7 (8.3)	−5.4 (9.9)	0.0 (4.7)	−4.5 (12.4)	6.8 (3.7)	−2.3 (10.7)	4.5 (13.6)	8.9 (17.7)	6.7 (6.3)	−4.4 (4)	6.7 (0)
	High QC	−1.5 (7.9)	0.0 (2.2)	3.0 (4.5)	8.9 (2)	−1.1 (3.5)	1.7 (11.1)	−1.7 (7.6)	−1.1 (11.2)	4.3 (3.5)	−4.3 (6.7)	0.0 (3.7)	2.1 (6.3)
**Carbamazepine**	Low QC	−7.7 (6.3)	0.0 (3.3)	−1.9 (10.2)	0.0 (3.3)	−1.8 (15.5)	8.8 (2.8)	1.8 (11.9)	12.3 (14.3)	7.8 (15.7)	6.3 (6.7)	−6.3 (0)	1.6 (2.7)
	High QC	4.4 (7)	3.4 (3.7)	7.8 (3.6)	13.6* (2.6)	1.4 (4.5)	−0.4 (6.2)	−1.1 (7.3)	1.1 (8)	−3.1 (4.9)	−11.3 (2)	2.1 (0)	8.2 (7.6)
**Valproic acid**	Low QC	−6.4 (4.7)	−5.0 (14.7)	−0.3 (7.4)	−2.6 (4.3)	−5.5 (2.7)	−2.7 (2.7)	−2.9 (4.4)	4.8 (10.7)	0.2 (8.5)	13.1 (1.6)	−0.9 (7.5)	7.0 (1.8)
	High QC	12.8	0.1	−1.5	5.8	2.1	3.2	2.7	2.1	1.7	−5.2	−5.0	5.2

		(7.6)	(6.3)	(6.4)	(10.1)	(4.6)	(7.4)	(5.2)	(3.3)	(8.3)	(3.3)	(9.5)	(0.8)
**Phenobarbital**	Low QC	3.9 (3.3)	2.0 (6.7)	0.0 (5.9)	7.8 (12.6)	–3.3 (10.6)	6.6 (5.3)	–1.6 (10)	1.6 (7.4)	–3.3 (10.6)	6.6 (5.3)	–1.6 (10)	1.6 (7.4)
	High QC	–5.2 (8.1)	–0.9 (3.3)	4.8 (10.7)	11.8 (5.3)	–5.2 (8.1)	–0.9 (3.3)	4.8 (10.7)	11.8 (5.3)	5.5 (10.8)	2.2 (3.2)	–3.3 (2)	12.1 (7.8)
**Phenytoin**	Low QC	–9.6 (4.2)	–2.9 (9.9)	–11.8 (10.9)	6.6 (12.6)	–7.5 (11.9)	0.7 (0)	0.0 (3.4)	–6.0 (8.6)	12.5 (9)	12.5 (10.4)	0.0 (5.6)	2.2 (3.3)
	High QC	3.9 (3.5)	7.8 (5.5)	–1.6 (7)	11.7 (3)	–1.2 (12.3)	0.6 (6.4)	–9.5 (10.8)	0.6 (8)	–0.5 (12)	–12.7 (19.1)	0.0 (0.8)	–3.4 (6.1)

Volume effect study. Expressed as % difference of the tested volumes compared to the reference QCs (35 μL)
*Statistically significant difference from 35μL reference QCs (one-way ANOVA with Bonferroni post hoc analysis. $p<0.05$) ($n = 3$).

Table S6. DBS homogeneity study

Analyte		Hct 0.2	Hct 0.4	Hct 0.6
**Levetiracetam**	Low QC	1.8	−9.4	−0.4
	High QC	7.1	−2.8	2.3
**Lacosamide**	Low QC	8.9	−8.3	0.0
	High QC	9.5	1.7	6.8
**Lamotrigine**	Low QC	−6.0	−6.8	1.6
	High QC	−1.2	−2.3	7.7
**10,11-Dihydro-10-hydroxy-carbamazepine**	Low QC	2.2	−7.6	0.5
	High QC	2.9	−0.8	3.6
**Carbamazepine-10,11-epoxide**	Low QC	3.7	−9.1	2.9
	High QC	8.5	−1.2	8.3
**Oxcarbazepine**	Low QC	8.1	−4.3	0.0
	High QC	5.4	−3.9	2.1
**Carbamazepine**	Low QC	7.7*	−7.8	4.6
	High QC	5.9	0.0	0.0
**Valproic acid**	Low QC	−0.5	−1.3	−3.2
	High QC	3.6	−1.4	−0.6
**Phenobarbital**	Low QC	13.2	0.0	−1.5
	High QC	−1.1	2.2	−9.7
**Phenytoin**	Low QC	−1.4	−2.3	−4.2
	High QC	18.5	−3.7	4.0

DBS homogeneity study. Expressed as %difference of the lateral punches from the standardized QCs (central punches).
*Statistically significant different from the central reference QCs (two-sided t test. $p<0.05$) ($n = 3$).

Table S7. Ratios between plasma and blood, VAMS or DBS concentrations from paired samples from patients under AEDs treatment

Analyte	Paired samples (n)	Blood/plasma ratio (%CV)	VAMS/plasma ratio (%CV)	DBS/plasma ratio (%CV)	VAMS/DBS ratio (%CV)
**VPA**	37	0.83 (11.1)	0.94 (9.3)	0.95 (13.9)	1.01 (18.5)
**LEV**	23	0.71 (27.7)	1.04 (21.1)	1.33 (34)	0.82 (20)
**LMT**	11	1.15 (30.8)	0.92 (13.7)	1.33 (44.4)	0.76 (22.2)
**DHCB**	11	0.99 (13.9)	0.98 (4.9)	1.12 (16.8)	0.89 (14.7)
**CBZ**	7	1.17 (23.6)	0.93 (7.1)	1.07 (25.3)	0.88 (20.9)
**LA**	4	0.61 (40.7)	0.8 (32.3)	0.84 (38.5)	1.11 (50.9)
**PHB**	2	1.76 (1.4)	0.79 (2.0)	0.78 (20.2)	1.05 (18.3)
CBZ: Carbamazepine; DHCB: 10,11-Dihydro-10-hydroxy-carbamazepine; LA: Lacosamide; LEV: Levetiracetam; LMT: Lamotrigine; PHB: Phenobarbital; VPA: Valproic acid					

Supplementary figure

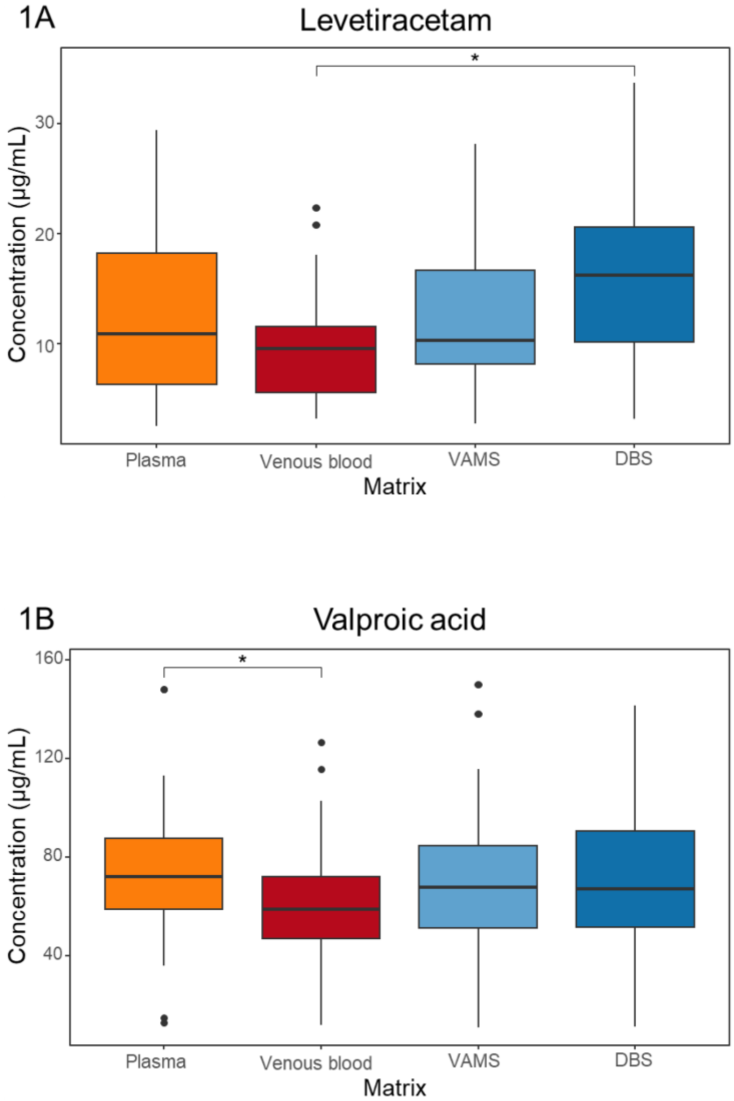

Figure S1. Levetiracetam (1A) and valproic acid (1B) concentrations in paired real samples. Significant differences between matrices according to a Wilcoxon test are indicated with an asterisk.
